# Supplementary material for: Efficacy of Minnelide in a Next-Generation Dual-Recombinase Regulated Genetically Engineered Mouse Model of CIC::DUX4 Sarcoma
Source: bioRxiv. 2025 Nov 8:2025.11.06.687065. Preprint. [Version 1] doi: 10.1101/2025.11.06.687065 (PMC12637584; doi:10.1101/2025.11.06.687065)
Supplement: Supplement 1 [file media-1.pdf]

Supplementary Figure 1

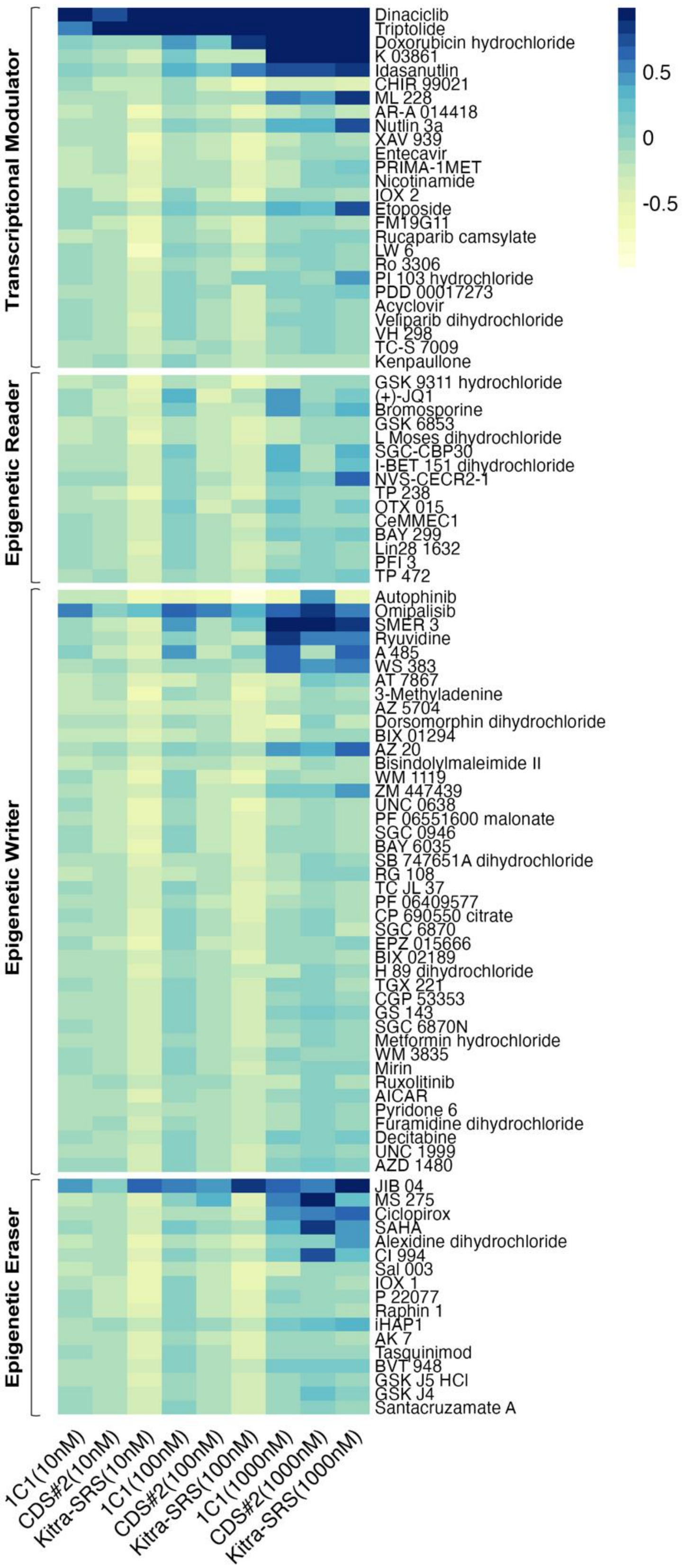

Figure S1. Viability drug screen on human CDS cells using the Tocriscreen Epigenetics 3.0 compound library. Top 100 compounds at 10nM, 100nM and 1000nM concentrations.

Supplementary Figure 2

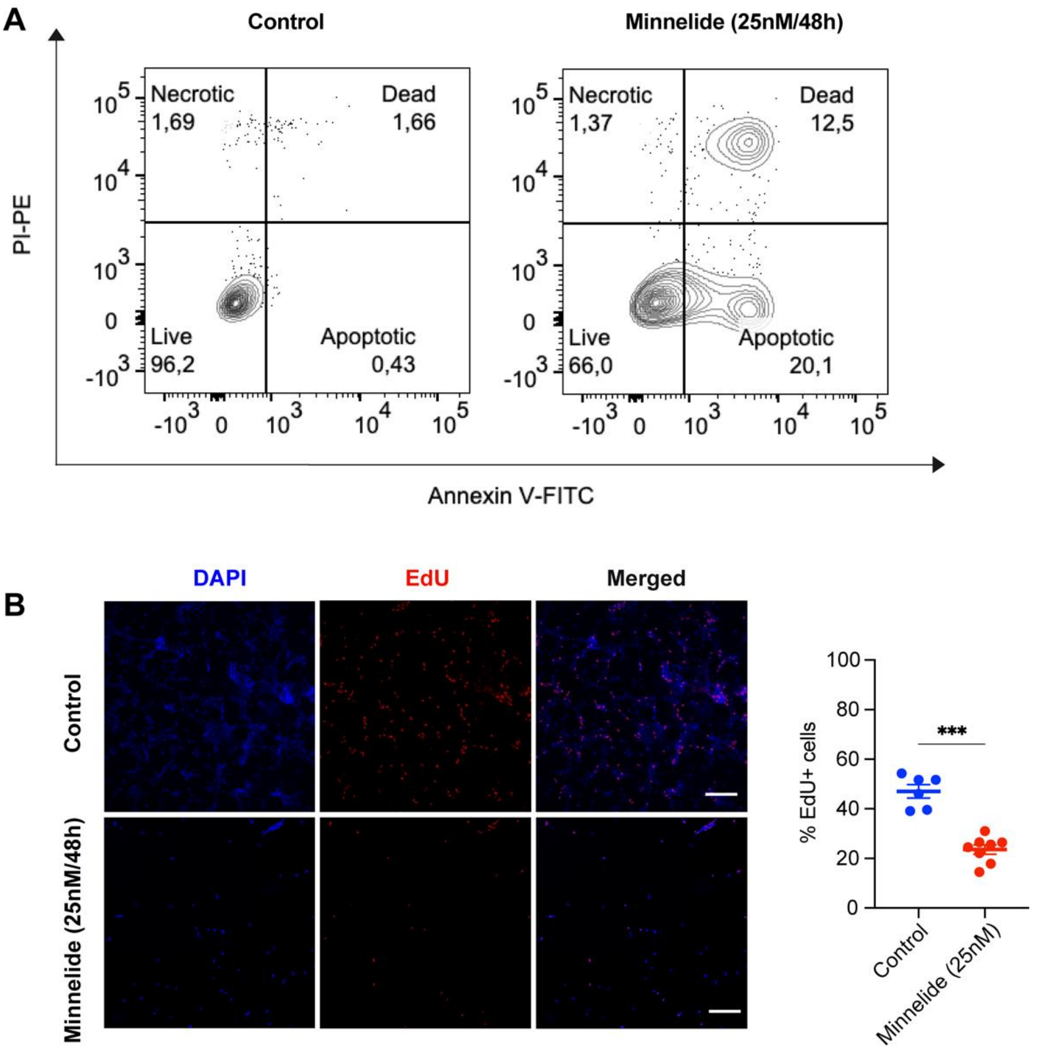

Figure S2. A. Annexin V/PI flow cytometry on ECD1 CDS cells treated with Minnelide for 48h. **B.** EdU labeling on human ECD1 CDS cells treated with Minnelide for 48h. ***P ≤ 0.001.

Supplementary Figure 3

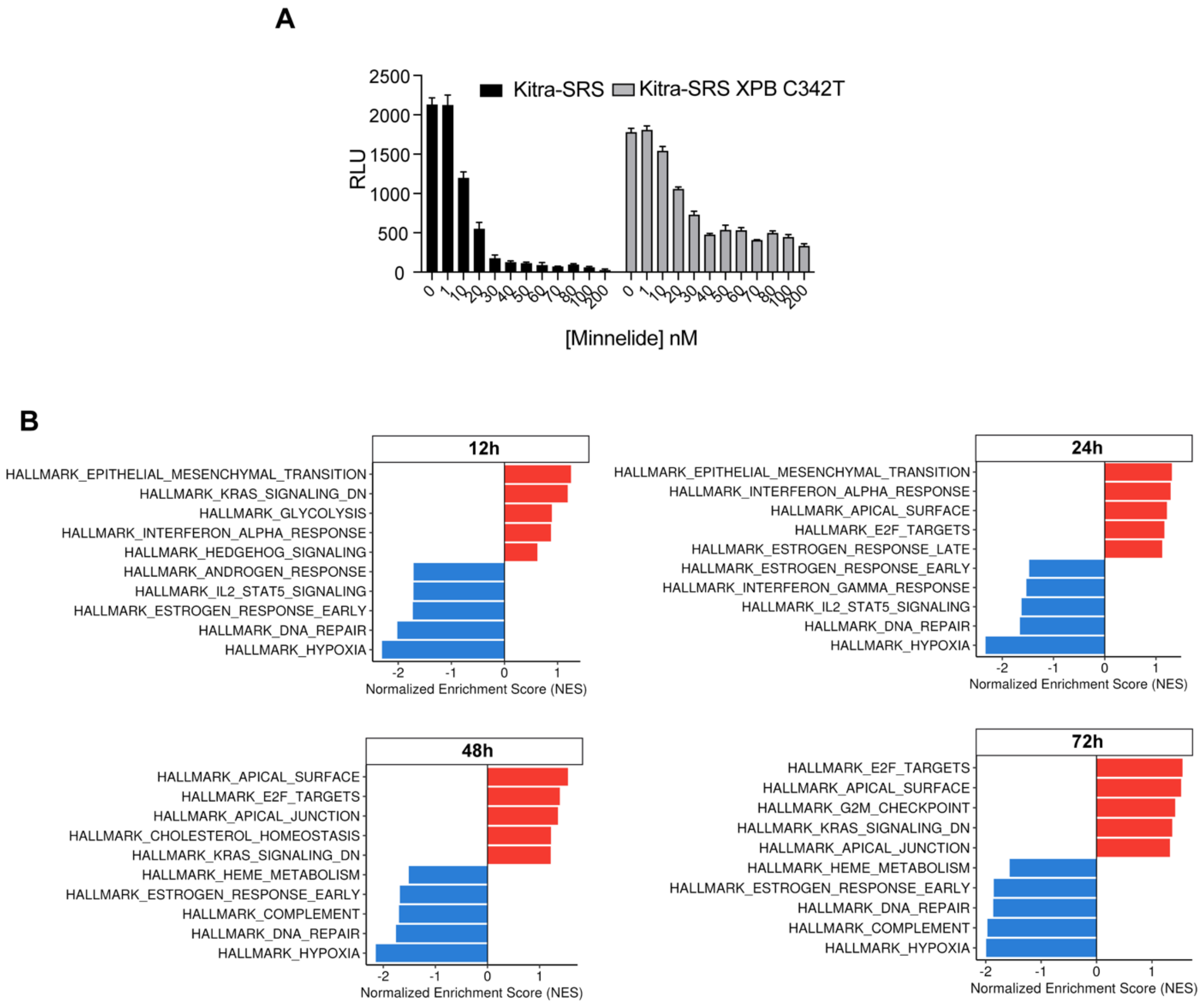

Figure S3. A. Cell-titer-glo assay demonstrates human CDS (Kitra-SRS) cells expressing XPB C342T are resistant to Minnelide. **B.** GSEA pathway analysis on Kitra-SRS cells treated with 25nM Minnelide for 12h, 24h, 48h and 72h

Supplementary Figure 4

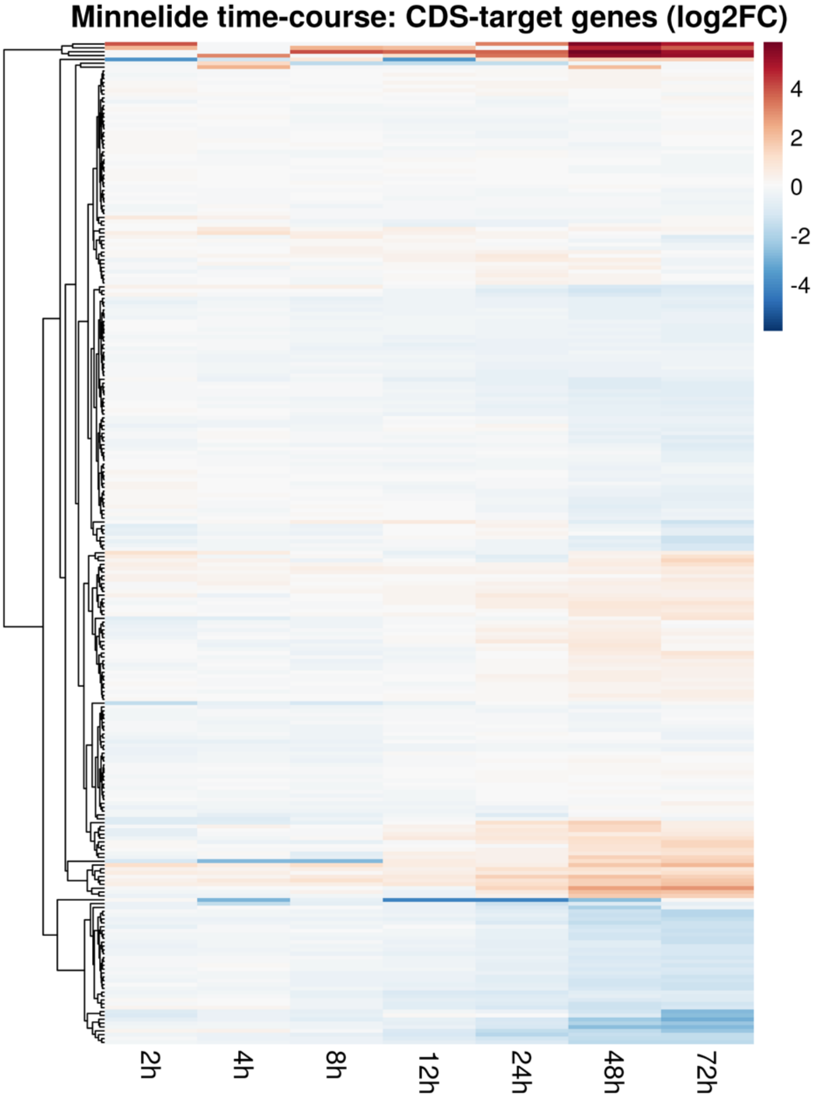

Figure S4. Heatmap showing expression of CIC::DUX4 target genes following Minnelide treatment for 2, 4, 8, 12, 24, 48, and 72 hours. The majority of CDS target genes, including *ETV1/4/5*, remained largely unchanged until 72 hours post-treatment.
